# Protein C Mutation (A267T) Results in ER Retention and Unfolded Protein Response Activation

**DOI:** 10.1371/journal.pone.0024009

**Published:** 2011-08-25

**Authors:** Lena Tjeldhorn, Nina Iversen, Kirsten Sandvig, Jonas Bergan, Per Morten Sandset, Grethe Skretting

**Affiliations:** 1 Department of Haematology, Oslo University Hospital, Oslo, Norway; 2 Medical Genetics, Oslo University Hospital, Oslo, Norway; 3 Department of Biochemistry, Institute for Cancer Research, Oslo University Hospital, Oslo, Norway; 4 Faculty of Medicine, Clinic of Specialized Medicine and Surgery, Institute of Clinical Medicine, University of Oslo, Oslo, Norway; 5 Faculty of Medicine, Centre for Cancer Biomedicine, Institute of Clinical Medicine, University of Oslo, Oslo, Norway; 6 Department of Molecular Biosciences, Faculty of Mathematics and Natural Sciences, University of Oslo, Oslo, Norway; Karolinska Institutet, Sweden

## Abstract

**Background:**

Protein C (PC) deficiency is associated with a high risk of venous thrombosis. Recently, we identified the PC-A267T mutation in a patient with PC deficiency and revealed by in vitro studies decreased intracellular and secreted levels of the mutant. The aim of the present study was to characterize the underlying mechanism(s).

**Methodology/Principal Findings:**

CHO-K1 cells stably expressing the wild-type (PC-wt) or the PC mutant were generated. In order to examine whether the PC mutant was subjected to increased intracellular degradation, the cells were treated with several inhibitors of various degradation pathways and pulse-chase experiments were performed. Protein-chaperone complexes were analyzed by treating the cells with a cross-linker followed by Western blotting (WB). Expression levels of the immunoglobulin-binding protein (BiP) and the phosphorylated eukaryotic initiation factor 2α (P-eIF2α), both common ER stress markers, were determined by WB to examine if the mutation induced ER stress and unfolded protein response (UPR) activation. We found no major differences in the intracellular degradation between the PC variants. The PC mutant was retained in the endoplasmic reticulum (ER) and had increased association with the Grp-94 and calreticulin chaperones. Retention of the PC-A267T in ER resulted in UPR activation demonstrated by increased expression levels of the ER stress markers BiP and P-eIF2α and caused also increased apoptotic activity in CHO-K1 cells as evidenced by elevated levels of DNA fragmentation.

**Conclusions/Significance:**

The reduced intracellular level and impaired secretion of the PC mutant were due to retention in ER. In contrast to other PC mutations, retention of the PC-A267T in ER resulted in minor increased proteasomal degradation, rather it induced ER stress, UPR activation and apoptosis.

## Introduction

Activated protein C (PC) is a vitamin K-dependent plasma glycoprotein that plays an important role in the regulation of blood coagulation [1]. PC deficiency is caused by mutations in the gene encoding PC, and is clinically associated with increased risk of venous thrombosis [2]. At present, nearly 200 various mutations in the PC gene have been described [3] and the functional effects of several PC mutations shown to be associated with PC deficiency have previously been studied *in-vitro* [4]–[11]. The majority of the missense mutations in PC lead to protein misfolding and consequently to retention of the mutants in the endoplasmic reticulum (ER) with subsequent degradation by proteasomes in a process called ER associated degradation (ERAD) [11]–[13].

PC is synthesized in liver cells where it is subjected to several posttranslational modifications in the ER and in the Golgi apparatus [14]. The processing of proteins in ER is controlled by chaperones, which facilitate protein folding and ensure that only correctly folded proteins are transported from the ER to Golgi [15]. Accumulation of misfolded proteins in ER can disturb homeostasis and result in ER stress, which activates the cellular unfolded protein response (UPR). This response eliminates ER stress by increasing the efficiency of protein folding, promoting ERAD and attenuating protein synthesis of mutated proteins [16]. Up-regulated expression of chaperones has been demonstrated in several studies on mutated proteins in general [17], [18]. A majority of the reported misfolded glycoproteins, including some mutated PC variants [11]–[13], are retrotranslocated across the ER membrane and degraded by ERAD. Some of the other mutant proteins are degraded by other proteases found in the ER and in the cytosol [19]–[21]. However, a few studies have described misfolded proteins, which were retained in the ER for a longer period of time without being degraded at all. These proteins were accumulated in the ER and led to elevated ER stress evidenced by increased expression levels of proteins such as the immunoglobulin-binding protein (BiP), the protein kinase-like ER kinase (PERK), and the CCAAT/enhancer-binding protein homologous protein (CHOP), all common markers of ER stress and UPR activation [17], [18], [22]. It has been shown that accumulation of misfolded proteins in the ER was associated with activation of PERK resulting in phosphorylation of the eukaryotic initiation factor 2α (eIF2α) with subsequent down-regulation of the protein synthesis [22]–[24]. Prolonged ER stress and UPR activation are associated with ERAD dysfunction, cell injury and apoptosis contributing to pathogenesis of many diseases [17], [22], [23], [25]–[27].

In a recent study [28], we found that both the intra- and extracellular levels of the PC-A267T mutant were strongly reduced compared to the wild-type PC (PC-wt) in transiently transfected cells despite the fact that there were no differences in the mRNA level. The aim of the present study was to obtain further insight into potential mechanisms of PC deficiency caused by the A267T mutation using stably transfected cells. We demonstrate that the A267T mutation caused retention of the PC molecule in the ER, most probably due to increased association with chaperones. In contrast to what has been reported for other PC mutants, the PC-A267T was only slightly subjected to proteasomal degradation, rather it induced ER stress, UPR activation and apoptosis in the cells.

## Materials and Methods

### Cell culture and stable transfection

Chinese hamster ovary cells (CHO-K1, CCL-61, American Type Culture Collection, Rockville, MD, USA) was maintained in Dulbecco's Modified Eagles Medium (DMEM, Cambrex BioScience, Verviers, Belgium) supplemented with 10% fetal bovine serum (FBS), 100 units/ml penicillin, 100 µg/ml streptomycin (Biowhittaker (TM), Luna, Belgium) and 5 µg/ml vitamin K1 (Sigma-Aldrich, St. Louis, MO, USA) at 37°C in humidified air with 5% CO_2_. The CHO-K1 cells were transfected with cDNAs for PC-wt or PC-A267T cloned into the pcDNA3.1/V5-His-TOPO expression vector (Invitrogen Carlsbad, CA, USA) as previously described [28]. The neomycin resistance gene in the pcDNA3.1/V5-His-TOPO vector allows for selection of cells with stable expression of PC variants. The transfected cells were grown in complete DMEM supplemented with 800 µg/ml of geneticin (G-418, Invitrogen) for three weeks. Stably transfected clones were isolated and expanded. Clones expressing high PC levels were selected for further experiments. The cell lines were maintained in complete DMEM containing 400 µg/ml of G-418.

### Effect of protein degradation inhibitors on PC-wt and PC-A267T biosynthesis

Stably transfected CHO-K1 cells were grown in 12-well plates until 60–80% confluence was reached. The cells were incubated with DMEM/1% FBS containing either 10 µM lactacystin (LCT, Sigma-Aldrich), 50 mM ammonium chloride (NH_4_Cl, Sigma-Aldrich), 50 µM N-acetyl-Leu-Leu-Norleucinal (ALLN, Biomol, Enzo Life Science, Farmingdale, NY, USA), 50 µM E-64-d (BioMol), 75 µM AAF-CMK (BioMol) or 100 nM bafilomycin (BFM, Sigma-Aldrich) for 8 h or 24 h. The concentration of PC antigen in cell lysates and culture medium were measured using the Elisara Protein C Kit (Aniara Corporation). The total protein concentrations in the cell lysates were measured by the BCA™ Protein Assay kit (Pierce, Rockford, IL, USA). PC antigen levels in cell lysates or culture medium were normalized against the total protein concentrations of the corresponding lysate samples. To examine the overall degradation rate of the PC variants the cells were washed twice with methionine-deficient medium and cultured for 30 min in methionine-deficient medium containing 50 µCi/ml [^35^S]methionine (Perkin Elmer, Boston, MA, USA). The cells were then cultured in complete DMEM supplemented with vitamin K1 for 0, 1.5, 3 and 6 h. The cell extracts were lysed with RIPA buffer supplemented with protease inhibitor cocktail (Sigma-Aldrich). The [^35^S]methionine-labeled PC was immunoprecipitated from cell extracts using a polyclonal antibody against PC (Aniara) and Dynabeads (Invitrogen). The immunoprecipitates were separated by SDS-PAGE and transferred to a polyvinylidene difluoride (PVDF) membrane (Bio-Rad) and the bands were visualized using autoradiography. The intensities of the bands were quantified using ImageJ software (National Institute of Health, USA).

### Cross-linking of proteins and Western blot analysis

Stably transfected CHO-K1 cells were incubated with 1.5 mM dithiobis[succinimydylpropionate] (DSP, Pierce) for 30 min on ice and quenched with 100 mM Tris-HCl (pH 7.6) for 20 min before lysis in RIPA buffer (Sigma-Aldrich). Cell lysates were run on 7.5% Tris-HCl gels (Bio-Rad, Hercules, CA, USA) at 120 V for 95 min. The proteins were electrotransferred onto a PVDF membrane (Bio-Rad) and blocked in 5% nonfat dry milk in TBS/0.1% Tween (Bio-Rad). The membranes were incubated with primary rabbit polyclonal anti-PC (Aniara Corporation) followed by incubation with the appropriate horseradish peroxidase (HRP)-conjugated secondary antibody (Santa Cruz Biotechnology, Santa Cruz, CA, USA). To identify chaperones, lysates from cells treated with DSP were separated on SDS-PAGE and immunoblotted with rabbit monoclonal anti-BiP and anti-calnexin, rabbit polyclonal anti-Grp-94, anti-calreticulin, anti-ERp57 and anti-PDI (all from Cell Signaling, Beverly, MA, USA). Proteins were visualized using the Amersham™ ECL Plus Western Blotting Detection System (GE Healthcare, Buckinghamshire, UK). Images were produced using the Luminescent Image Analyzer LAS-4000 mini (Fujifilm, Tokyo, Japan).

### Low temperature and chemical chaperone analysis

CHO-K1 cells stably expressing PC-wt and PC-A267T were incubated at either 26°C or 37°C. The cells were also incubated with DMEM/1% FBS containing 2% dimethyl sulfoxide (DMSO) (Sigma-Aldrich). After two days the cell lysates and culture medium were harvested. PC antigen levels were determined as described above and normalized against the concentration of total protein in the corresponding lysate samples.

### Expression of proteins associated with ER stress and UPR

Stably transfected CHO-K1 cells were grown to 70–80% confluence. The cells were then lysed in ice-cold RIPA lysis buffer (Sigma-Aldrich) supplemented with protease and phosphatase inhibitor cocktails (Sigma-Aldrich). Cell lysates were analyzed on a 10% Tris-HCl gel (Bio-Rad) under reducing conditions. After electrophoresis, the proteins were electrotransferred to PVDF membranes (Bio-Rad) and immunoblotted with rabbit polyclonal anti-phospho-eIF2α (P-eIF2α), rabbit polyclonal anti-eIF2α, rabbit monoclonal anti-BiP (all from Cell Signaling Technology) or mouse monoclonal anti-α-tubulin (Sigma-Aldrich). The membranes were incubated with appropriate HRP-conjugated secondary antibodies (Santa Cruz Biotechnology) and visualized using the Amersham™ ECL Plus Western Blotting Detection System (GE Healthcare). Images were produced using the Luminescent Image Analyzer LAS-4000 mini (Fujifilm, Tokyo, Japan), and the Multi Gauge Ver3.X software program (Microsoft Corporation, USA) was used to quantify the intensity of the bands.

### Analysis of apoptosis

The levels of cytoplasmic histone-bound DNA fragments in cells expressing PC-wt and PC-A267T were measured using the Cell Death Detection ELISA^PLUS^ kit (Roche Applied Science, Indianapolis, IN, USA). The DNA fragmentation assay was performed according to the manufacturer's recommendation with minor modifications. Cells were seeded in six-well plates and grown to approximately 80% confluence whereafter they were lysed in NP-40 buffer (10 mM Tris-HCl, 150 mM NaCl, 1% NP-40). The levels of cytoplasmic histone-associated-DNA fragments (mono- and oligonucleosomes) were determined photometrically using SPECTRAmax PLUS^384^ Microplate Spectrophotometer (Molecular Devices, LLC, Sunnyvale, CA USA).

### Statistics

All results were tested for statistical significance with the non-parametric Mann-Whitney test and p values ≤ 0.05 were considered statistically significant. GraphPad Prism version 5 (GraphPad Software Inc., San Diego, CA, USA) was used for statistical analysis.

## Results

We have previuosly found, using transient transfected cells, that the intracellular and secreted PC-A267T levels were reduced compared to PC-wt, and the majority of the PC mutant was localized in ER [28]. The reduced levels and retention of PC-A267T were confirmed in the stably transfected cells (data not shown).

### No increased intracellular degradation of PC-A267T

**Figure 1:**
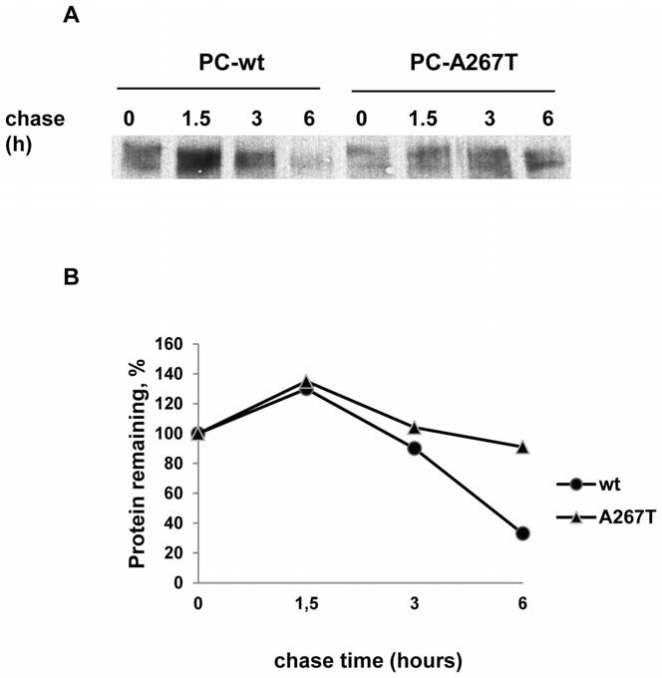
Pulse–chase analysis of intracellular PC-wt and PC-A267T in stably transfected CHO-K1 cells. Stably transfected CHO-K1 cells were pulse-labeled for 30 min with [^35^S]methionine and chased for 0, 1.5, 3 and 6 h. Equivalent amounts of cell extracts were immunoprecipitated using a polyclonal antibody against PC. Immunoprecipitates were analyzed by SDS-PAGE under reducing conditions (A). The amount of radioactivity remaining at each time point was plotted as the percentage of that measured in the immunoprecipate from unchased cells. The mean values from two independent experiments are presented. The turnover rates of the intracellular PC-wt and PC-A267T are depicted as • and ▴, respectively (B).

In a recent report we showed a slight increase in the proteasomal degradation of the PC-A267T variant using LCT, while the lysosomal degradation was unaffected as assessed by BFM [28]. These findings were confirmed in the stable cell lines (data not shown). To expand the knowledge on the intracellular degradation, the two stable cell lines were treated with additional inhibitors. Treatment with ALLN, which is a less specific proteasomal inhibitor, did not indicate any significant difference in the degradation between the two PC molecules (7.3% of PC-wt protection versus 8.1% of PC-A267T, ns). NH_4_Cl, an inhibitor of lysosomal degradation, indicated a slightly increased degradation of PC-wt compared to PC-A267T (p<0.0001). Treatment of the cells with AAF-CMK and E-64-d led to non-significant changes of the PC levels of both variants indicating no degradation by the cytosolic protease tripeptidyl peptidase II or calpain, respectively (Table 1). The possibility of increased intracellular degradation of PC-A267T was further investigated by pulse-chase experiments. The two cell lines were pulsed with radioactive [^35^S] methionine for 30 min and chased for up to 6 h. PC was immunoprecipitated from the lysates and the turnover rate of the two PC variants were determined by quantifying the density of PC bands. As shown in Figure 1, the total amount of radioactivity after 6 h for PC-wt and PC-A267T were decreased by 91% and 33%, respectively, indicating that the PC mutant was more stable than the PC-wt. As seen in Figure 1, the amount of the PC mutant was reduced compared to the PC-wt when the chase started. This is probably not due to reduced synthesis rate rather a difference in the PC mRNA levels in the stably transfected cells since the cells expressing the PC-A267T only had a third of the PC mRNA level in the PC-wt expressing cells.

**Table 1:** Effect of protein degradation inhibitors on PC levels.

Treatments	PC-wt,%	PC-A267T,%
Control	100	100
ALLN (50 µM), n = 9	107.3±7.4	108.1±7.2
NH_4_Cl (50 mM), n = 9	122.7±2.2	100.8±2.8*
AAF-CMK (75 µM), n = 9	95.1±3.2	92.8±2.4
E-64-d (50 µM), n = 9	94.1±2.2	87.6±2.9

CHO-K1 cells stably expressing PC-wt or PC-A267T were incubated with medium containing ALLN, NH_4_Cl, AAF-CMK and E-64-d for 24 or 8 h. PC levels were measured in cell lysates and culture medium and results are calculated as percentage of corresponding results from untreated cells. Data are presented as mean ± SEM.

*(p≤0.05, Mann-Whithey test), PC-A267T versus PC-wt.

### Stronger association of chaperones with PC-A267T

**Figure 2:**
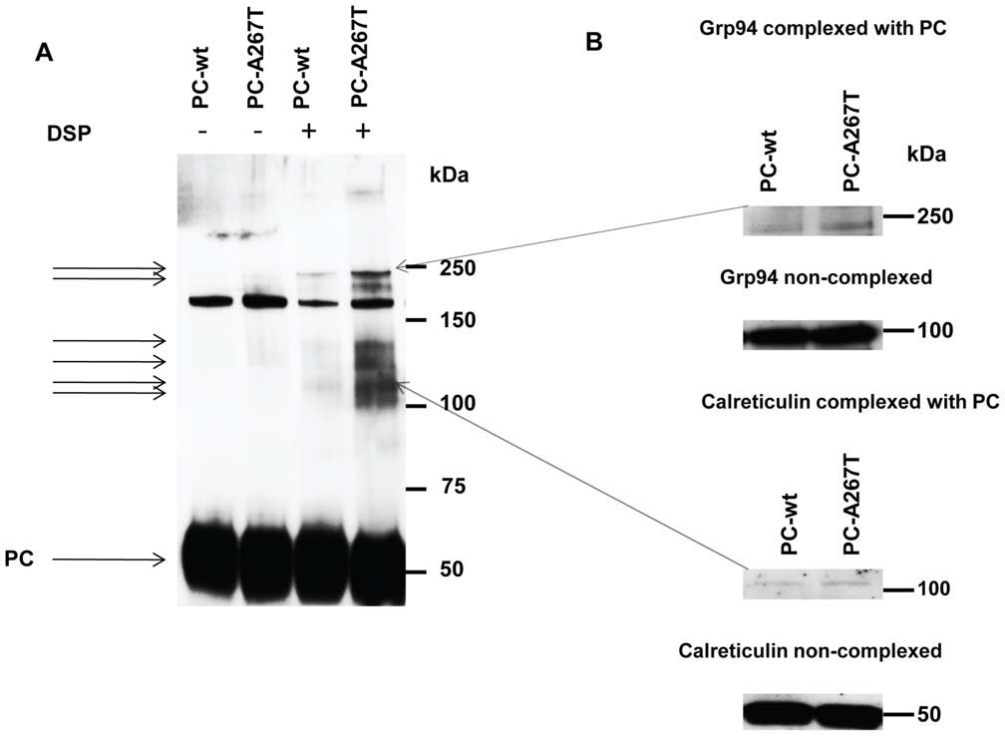
Identification of PC-chaperone complexes in CHO-K1 cells stably expressing PC-wt or PC-A267T. The cells were pretreated with dithiobis[succinimydylpropionate] (DSP) before cell lysis. Cell lysates with equivalent amounts of PC were separated on SDS-PAGE under reducing conditions and immunoblotted with anti-PC to detect PC-chaperone complexes. Similar results were obtained in three independent experiments. The molecular weight standards are shown on the right (kDa) and the positions of the PC-chaperone complexes are marked with the arrows on the left (A). Identification of chaperones associated with the PC mutant. The cell lysates pretreated with DSP were subjected to SDS-PAGE and Western blot analysis with antibodies against indicated chaperones. Two independent experiments with similar results were performed. (B).

To examine whether PC-A267T was retained in ER due to an excessive binding of chaperones, we analyzed PC-chaperone complexes in cells expressing PC-wt or PC-A267T. Since intracellular protein-chaperone complexes are unstable, the cells were treated with the cross-linker DSP before lysis in order to minimize the release of chaperones from PC. Equal amounts of PC (as measured by ELISA) were analyzed. PC appeared as a band with a molecular weight of approximately 60 kDa (Figure 2A). Several PC-chaperone complexes with molecular weights of approximately 105, 115, 130, 140, 225, and 250 kDa were detected in the lysates of cells treated with DSP, but not in control cells. The prominent band at around 170 kDa represents unspecific band as it was also detected in lysates from cells not expressing PC (data not shown). The intensity of the bands representing PC-chaperone complexes from cells expressing PC-A267T was stronger compared to the corresponding bands from PC-wt expressing cells indicating an excessive binding of chaperones to PC-A267T. Immunoblotting analysis using antibodies against several chaperones revealed that PC was associated with calreticulin (55 kDa) and Grp-94 (94 kDa) (Figure 2B) in the bands with molecular weights of approximately 115 and 250 kDa, respectively. The size of the band representing PC associated with Grp-94 indicated that two Grp-94 molecules might be bound to one PC molecule. Grp-94 and calreticulin were associated with both PC variants, however, quantification of the bands showed stronger association of these chaperones with PC-A267T (approximately 50% and 20% increased association for Grp-94 and calreticulin, respectively). No bands representing PC in complex with BiP, PDI, calnexin or Erp57 could be detected.

### No rescue of PC-A267T by low temperature and chemical chaperone

**Figure 3:**
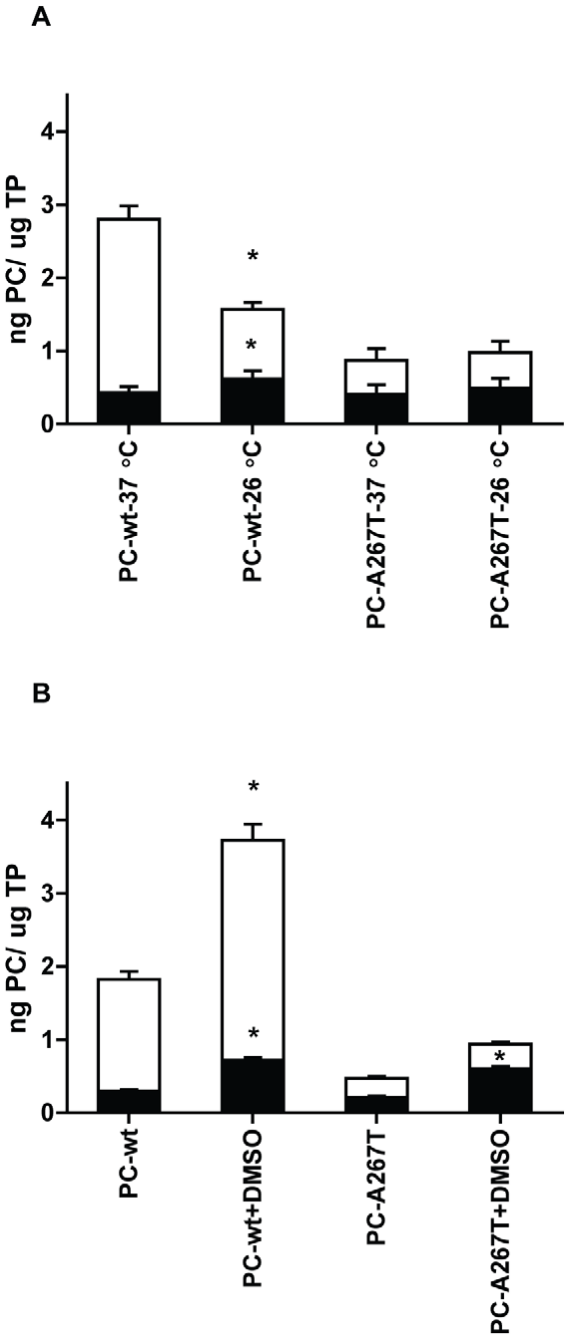
Effect of low temperature and DMSO on PC biosynthesis. CHO-K1 cells stably expressing PC-wt or PC-A267T were incubated at 26°C (A) or in culture medium containing 2% DMSO (B). PC concentrations were quantified in cell lysates (▪) and culture medium (□), and normalized to total protein (TP) concentrations of the corresponding lysate samples. Histograms and the bars represent mean + SEM values, n = 9. * (p≤0.05, Mann-Whithey test), PC-wt/PC-A267T treated versus PC-wt/PC-A267T untreated.

Growing cells expressing protein mutants at reduced temperature or in the presence of chemical chaperones can improve correct folding of misfolded proteins and thereby enhance intracellular transport and secretion [29]–[32]. To examine whether a potential folding defect of PC-A267T could be corrected by reduced temperature or DMSO, the cells were grown at 26°C or in the presence of 2% DMSO. The intracellular levels of both PC variants were increased in cells grown at 26°C compared to 37°C with a slightly larger increase of the PC-wt level compared to PC-A267T (Figure 3A). Incubation at 26°C strongly reduced the secretion level of the PC-wt, while the secretion level of PC-A267T was virtually unaffected. When the cells were treated with DMSO, the intracellular and secreted levels of both PC variants were increased to some extent compared to untreated cells (Figure 3B).

### PC-A267T induces ER stress, UPR activation and apoptosis

**Figure 4:**
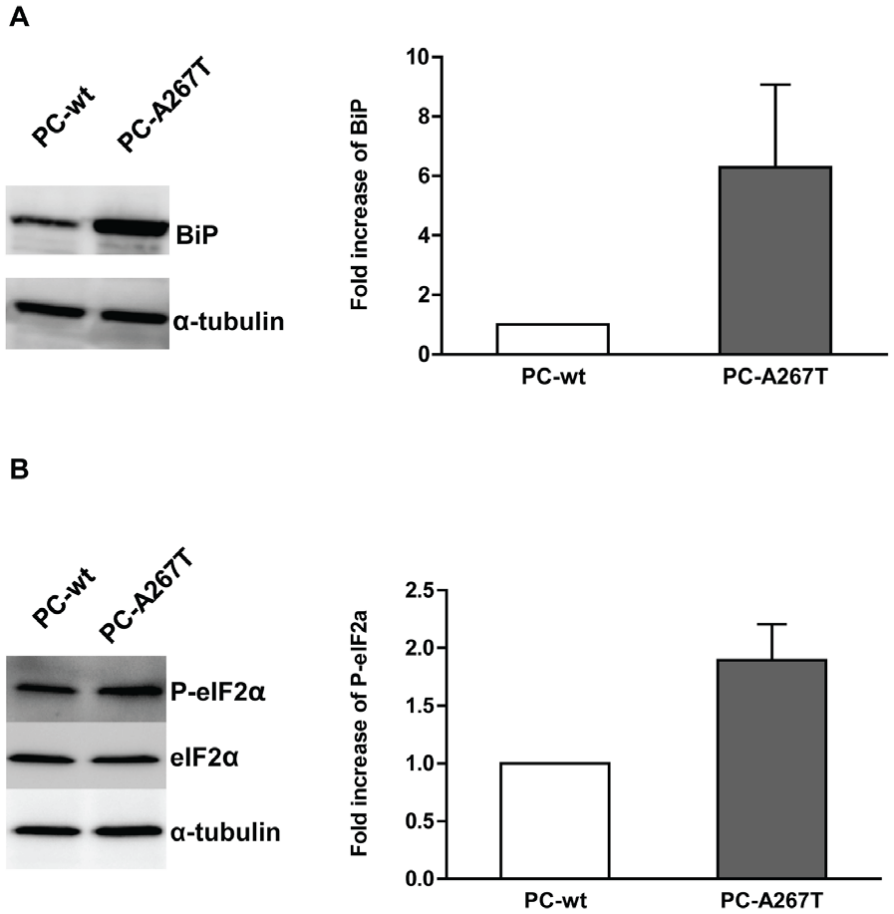
Expression levels of ER stress and UPR activation markers. Immunoglobulin-binding protein (BiP) (A) and phosphorylated eukaryotic initiation factor 2α (P-eIF2α) (B) levels were analyzed by Western blotting in lysates from CHO-K1 cells stably expressing PC-wt (lane 1) or PC-A267T (lane 2). Fold increase of BiP and P-eIF2α levels in CHO-K1 cells expressing PC-A267T are shown as histograms and bars, which represent mean + SEM values of expression levels detected in three independent experiments. BiP bands were quantified and normalized to corresponding amounts of α-tubulin. P-eIF2α bands were quantified and normalized to corresponding amounts of eIF2α.

**Figure 5:**
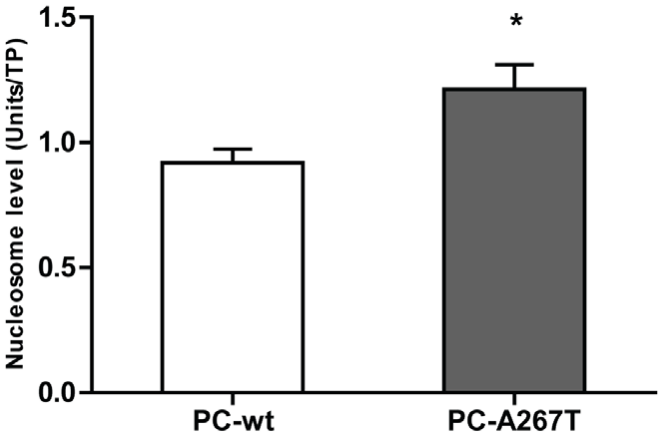
DNA fragmentation analysis in CHO-K1 cells stably expressing PC-wt or PC-A267T. The levels of cytoplasmic DNA fragments (mono- and oligonucleosomes) in cells stably expressing PC variants was measured by the Cell Death Detection ELISA^PLUS^ kit. The results were normalized to total protein (TP) concentrations and presented as mean + SEM values of four independent experiments (each experiment performed in triplicates). * (p≤0.05, Mann-Whithey test).

To investigate whether retention of the PC mutant in ER resulted in ER stress and UPR activation, we compared the expression levels of the ER stress markers BiP and P-eIF2α in cells expressing PC-wt or PC-A267T. In the cells expressing PC-A267T the level of BiP was strongly increased compared to cells expressing PC-wt (Figure 4A). Moreover, the P-eIF2α level in cells expressing PC-A267T was 1.6-fold increased relative to cells expressing PC-wt (Figure 4B). Prolonged ER stress and activation of the UPR may lead to apoptotic reactions in cells expressing mutated proteins. DNA fragmentation is one of the central indicators of apoptosis. The cytoplasmic mono- and oligonucleosome levels were significantly higher in cells expressing PC-A267T compared to cells expressing PC-wt indicating increased apoptotic activity in these cells (Figure 5).

## Discussion

The aim of the present study was to investigate the molecular basis of PC deficiency caused by the A267T mutation using CHO-K1 cells stably expressing the wild-type or PC mutant. Compared to PC-wt, which was located both in the ER and in the Golgi, PC-A267T was predominantly located in the ER indicating increased retention of the mutant. This was probably due to increased association with chaperones to the PC mutant. In contrast to other PC mutations studied in vitro, the retention of PC-A267T in the ER was not mainly associated with increased ERAD, rather it resulted in ER stress and UPR, which probably affected the PC biosynthesis.

Several missense mutations associated with PC deficiency have previously been studied in-vitro [4]–[11]. Some of these variants showed reduced secretion of PC into the culture medium due to their efficient degradation by ERAD [11]–[13]. The PC-A267T represents a different variant of a PC mutation based on the fact that although its secretion is severely impaired, the proteasomal degradation is only slightly elevated compared to the PC-wt and not sufficient to explain the reduced PC levels. Although proteasomal degradation is the most common pathway for the elimination of mutated proteins, several studies have described degradation of misfolded proteins by proteasome-independent pathways [19]–[21], [33]. In the present study, the results of the pulse-chase analysis and the treatment with several inhibitors of these alternative pathways gave no indications that the PC-A267T was degraded faster than the PC-wt. Thus, alternative mechanisms are most likely responsible for the reduced levels of this PC mutant.

Abnormalities in protein folding due to mutations have been attributed to the pathology of a number of diseases. Improper folded or misfolded proteins are recognized and retained by the folding chaperone machinery of the ER [15]. Confocal microscopy analysis indicated that PC-A267T was retained in the ER and analysis of PC-chaperone complexes suggested that the retention most likely was due to increased association of chaperones suggesting that PC-A267T could be misfolded. This is in agreement with previous studies, which demonstrated that mutations in coagulation factors such as factor VII [34], factor IX [20], and also PC [7], were associated with reduced protein secretion and prolonged association of the mutated proteins with chaperones in ER. In our study, we revealed that the PC mutant was associated with calreticulin and Grp-94, both well-known chaperones. Calreticulin interacts with N-linked glycoproteins and has several functions in the folding and assembling of these proteins. Grp-94 is a chaperone, which was found to be associated with several misfolded proteins, but its role in protein folding is not well understood. Although we found increased expression levels of BiP in cells expressing PC-A267T, no interaction of BiP with PC-A267T could be detected.

Increased binding of mutated proteins to chaperones in ER is a well documented phenomenon, and can result in either increased ERAD [15], [35] or increased folding efficiency of misfolded proteins [36]. The mechanism regulating the balance between folding and degradation of misfolded proteins is as yet not known. Several studies have, however, suggested that the destiny of misfolded proteins in the ER was dependent on the nature of the mutation and the chaperones available in the cells [37], [38]. Sekijima *et al.* [38] analyzed a large number of mutations in the transthyretin protein and concluded that the destiny of this protein was determined by the kinetic and the thermodynamic properties of the protein conformation caused by a mutation.

A variety of chemical chaperones, such as DMSO, and growing cells at low temperature, have been reported to efficiently improve the conformation of several misfolded proteins trapped in ER [29]–[32]. In our study, the secretion level of PC-A267T was not increased to the PC-wt level, neither by growing the cells at 26°C nor in the presence of DMSO. This indicates that the PC mutant did not adopt suitable conformation to be efficiently secreted. Based on these results it is impossible to establish that the PC-A267T mutant exhibits a folding defect that can be corrected by low temperature or DMSO. However, one can imagine that the PC-A267T mutation only causes a minor defect on the protein structure and that the ER quality machinery was unable to recognize the mutant protein as misfolded enough to direct it to ERAD. This hypothesis is in agreement with the interpretation of the A267T mutation obtained by a computational approach, as not being destructive on PC structure and function (http://www.itb.cnr.it/procmd/). Inefficient elimination of misfolded proteins by ERAD may lead to accumulation of mutated proteins in ER and several studies have shown that misfolded proteins, which were retained in ER without being efficiently degraded, activated ER stress and UPR [22], [23], [25], [26]. The main task of the UPR is to protect the cells from toxic effects of accumulated and aggregated mutated proteins in ER. The signaling cascade of UPR involves chaperone-assisted protein folding and increased transcription of genes encoding chaperones. One of the best characterized chaperone is BiP, which has a range of functions within the ER. BiP interacts with hydrophobic regions of misfolded proteins and facilitates folding, thus preventing aggregation in ER. In addition, BiP plays a central role in the regulation of UPR and elevated BiP levels are detected upon pathological stress responses [39]. In this study, we found increased expression level of BiP in cells expressing PC-A267T indicating that the retention of the mutant in ER induced ER stress and UPR activation. Another common effect of UPR activation is attenuation of mRNA translation in order to prevent influx of newly synthesized polypeptides in ER [22]–[24]. Although no evidence that the A267T mutation affected PC mRNA translation is provided in the present study, the increased level of P-eIF2α detected in cells expressing PC-A267T might indicate that the synthesis of PC was reduced as a result of UPR activation.

Despite initially beneficial effects of UPR in cells expressing misfolding-prone proteins, prolonged ER stress and UPR activation can lead to cell dysfunction and apoptotic cell death [16]. Several studies indicated that excessive UPR activation could affect normal cell functions, such as intracellular protein transport [40] and proteasomal degradation [25], [26]. In our study, the cells expressing the PC mutant had increased apoptotic activity as measured by elevated DNA fragmentation. In accordance with other studies, which reported that ER stress and UPR activation were the key events leading to functional impairment of cells producing the mutant proteins [18], [24]–[26], we suggest that the biosynthesis of PC-A267T was strongly impaired in the stably transfected CHO-K1 cells.

In summary, our study provides evidence that retention of the PC-A267T resulted in ER stress, UPR activation and apoptosis in CHO-K1 cells. This is in contrast to what has been reported for other PC mutants, which were degraded by the proteasomes. Thus, the present PC mutant represents the first example where proteasomal degradation is not the main mechanism for cells to manage mutant PC molecules.

## References

[1] Dahlback B, Villoutreix BO (2003). Molecular recognition in the protein C anticoagulant pathway.. J Thromb Haemost.

[2] Griffin JH, Evatt B, Zimmerman TS, Kleiss AJ, Wideman C (1981). Deficiency of protein C in congenital thrombotic disease.. J Clin Invest.

[3] D'Ursi P, Marino F, Caprera A, Milanesi L, Faioni EM (2007). ProCMD: a database and 3D web resource for protein C mutants.. BMC Bioinformatics.

[4] Sugahara Y, Miura O, Yuen P, Aoki N (1992). Protein C deficiency Hong Kong 1 and 2: hereditary protein C deficiency caused by two mutant alleles, a 5-nucleotide deletion and a missense mutation.. Blood.

[5] Sugahara Y, Miura O, Hirosawa S, Aoki N (1994). Compound heterozygous protein C deficiency caused by two mutations, Arg-178 to Gln and Cys-331 to Arg, leading to impaired secretion of mutant protein C.. Thrombosis and Haemostasis.

[6] Tokunaga F, Tsukamoto T, Koide T (1996). Cellular basis for protein C deficiency caused by a single amino acid substitution at Arg15 in the gamma-carboxyglutamic acid domain.. J Biochem.

[7] Katsumi A, Senda T, Yamashita Y, Yamazaki T, Hamaguchi M (1996). Protein C Nagoya, an elongated mutant of protein C, is retained within the endoplasmic reticulum and is associated with GRP78 and GRP94.. Blood.

[8] Lind B, Koefoed P, Thorsen S (2001). Symptomatic type 1 protein C deficiency caused by a de novo Ser270Leu mutation in the catalytic domain.. British Journal of Haematology.

[9] Lind B, Gedde-Dahl T, Tjonnfjord G, Villoutreix BO, Brosstad F (2002). Protein C deficiency caused by homozygosity for a novel PROC D180G mutation--in vitro expression and structural analysis of the mutation.. Thrombosis and Haemostasis.

[10] Naito M, Mimuro J, Endo H, Madoiwa S, Ogata K (2003). Defective sorting to secretory vesicles in trans-Golgi network is partly responsible for protein C deficiency: molecular mechanisms of impaired secretion of abnormal protein C R169W, R352W, and G376D.. Circulation Research.

[11] Zhou RF, Cai XH, Xie S, Wang XF, Wang HL (2006). Molecular mechanism for hereditary protein C deficiency in two Chinese families with thrombosis.. J Thromb Haemost.

[12] Nakahara M, Koyama T, Nakazawa F, Nishio M, Shibamiya A (2004). Gradually glycosylated protein C mutants (Arg178Gln and Cys331Arg) are degraded by proteasome after mannose trimming.. Thrombosis and Haemostasis.

[13] Nishio M, Koyama T, Nakahara M, Egawa N, Hirosawa S (2008). Proteasome degradation of protein C and plasmin inhibitor mutants.. Thrombosis and Haemostasis.

[14] Griffin JH, Fernandez JA, Gale AJ, Mosnier LO (2007). Activated protein C.. J Thromb Haemost.

[15] Trombetta ES, Parodi AJ (2003). Quality control and protein folding in the secretory pathway.. Annual Review of Cell and Developmental Biology.

[16] Malhotra JD, Kaufman RJ (2007). The endoplasmic reticulum and the unfolded protein response.. Seminars in Cell and Developmental Biology.

[17] Rebello G, Ramesar R, Vorster A, Roberts L, Ehrenreich L (2004). Apoptosis-inducing signal sequence mutation in carbonic anhydrase IV identified in patients with the RP17 form of retinitis pigmentosa.. Proc Natl Acad Sci U S A.

[18] Sorensen S, Ranheim T, Bakken KS, Leren TP, Kulseth MA (2006). Retention of mutant low density lipoprotein receptor in endoplasmic reticulum (ER) leads to ER stress.. J Biol Chem.

[19] Wakabayashi S, Yoshida H, Shigekiyo T, Koide T (2000). Intracellular degradation of histidine-rich glycoprotein mutants: tokushima-1 and 2 mutants are degraded by different proteolytic systems.. J Biochem.

[20] Enjolras N, Plantier JL, Rodriguez MH, Rea M, Attali O (2004). Two novel mutations in EGF-like domains of human factor IX dramatically impair intracellular processing and secretion.. J Thromb Haemost.

[21] Tsuda H, Tokunaga F, Nagamitsu H, Koide T (2006). Characterization of endoplasmic reticulum-associated degradation of a protein S mutant identified in a family of quantitative protein S deficiency.. Thrombosis Research.

[22] Nishitsuji K, Tomiyama T, Ishibashi K, Ito K, Teraoka R (2009). The E693Delta mutation in amyloid precursor protein increases intracellular accumulation of amyloid beta oligomers and causes endoplasmic reticulum stress-induced apoptosis in cultured cells.. Am J Pathol.

[23] Hashimoto Y, Tomiyama T, Yamano Y, Mori H (2003). Mutation (D472Y) in the type 3 repeat domain of cartilage oligomeric matrix protein affects its early vesicle trafficking in endoplasmic reticulum and induces apoptosis.. Am J Pathol.

[24] Park SY, Ye H, Steiner DF, Bell GI (2010). Mutant proinsulin proteins associated with neonatal diabetes are retained in the endoplasmic reticulum and not efficiently secreted.. Biochem Biophys Res Commun.

[25] Mulugeta S, Nguyen V, Russo SJ, Muniswamy M, Beers MF (2005). A surfactant protein C precursor protein BRICHOS domain mutation causes endoplasmic reticulum stress, proteasome dysfunction, and caspase 3 activation.. Am J Respir Cell Mol Biol.

[26] Uchio N, Oma Y, Toriumi K, Sasagawa N, Tanida I (2007). Endoplasmic reticulum stress caused by aggregate-prone proteins containing homopolymeric amino acids.. FEBS J.

[27] Liu Y, Wang Y, Wu C, Liu Y, Zheng P (2009). Deletions and missense mutations of EPM2A exacerbate unfolded protein response and apoptosis of neuronal cells induced by endoplasm reticulum stress.. Hum Mol Genet.

[28] Tjeldhorn L, Iversen N, Sandvig K, Bergan J, Sandset PM (2010). Functional characterization of the protein C A267T mutation: evidence for impaired secretion due to defective intracellular transport.. BMC Cell Biol.

[29] Vollrath D, Liu Y (2006). Temperature sensitive secretion of mutant myocilins.. Exp Eye Res.

[30] Yam GH, Roth J, Zuber C (2007). 4-Phenylbutyrate rescues trafficking incompetent mutant alpha-galactosidase A without restoring its functionality.. Biochem Biophys Res Commun.

[31] Gelsthorpe ME, Baumann N, Millard E, Gale SE, Langmade SJ (2008). Niemann-Pick type C1 I1061T mutant encodes a functional protein that is selected for endoplasmic reticulum-associated degradation due to protein misfolding.. J Biol Chem.

[32] Vu D, Di SC, Neerman-Arbez M (2008). Manipulating the quality control pathway in transfected cells: low temperature allows rescue of secretion-defective fibrinogen mutants.. Haematologica.

[33] Fayadat L, Siffroi-Fernandez S, Lanet J, Franc JL (2000). Degradation of human thyroperoxidase in the endoplasmic reticulum involves two different pathways depending on the folding state of the protein.. Journal of Biological Chemistry.

[34] Arbini AA, Mannucci M, Bauer KA (1996). A Thr359Met mutation in factor VII of a patient with a hereditary deficiency causes defective secretion of the molecule.. Blood.

[35] Vembar SS, Brodsky JL (2008). One step at a time: endoplasmic reticulum-associated degradation.. Nat Rev Mol Cell Biol.

[36] Susuki S, Sato T, Miyata M, Momohara M, Suico MA (2009). The Endoplasmic Reticulum-associated Degradation of Transthyretin Variants Is Negatively Regulated by BiP in Mammalian Cells.. J Biol Chem.

[37] Molinari M (2007). N-glycan structure dictates extension of protein folding or onset of disposal.. Nat Chem Biol.

[38] Sekijima Y, Wiseman RL, Matteson J, Hammarstrom P, Miller SR (2005). The biological and chemical basis for tissue-selective amyloid disease.. Cell.

[39] Hebert DN, Molinari M (2007). In and out of the ER: protein folding, quality control, degradation, and related human diseases.. Physiol Rev.

[40] Amodio G, Renna M, Paladino S, Venturi C, Tacchetti C (2009). Endoplasmic reticulum stress reduces the export from the ER and alters the architecture of post-ER compartments.. Int J Biochem Cell Biol.

